# Progress in Ophthalmology

**Published:** 1899-07-08

**Authors:** 


### PROGRESS IN OPHTHALMOLOGY.

**Ophthalmia Neonatorum.**—For the prevention of ophthalmia neonatorum Dr. Webster Fox[1] advocates very strongly the use of argent nitratis in 1 to 2 per cent. solution, and says a drop or two should be instilled even where there is no discharge from the mother. He says as this solution is mild and only produces a smarting sensation no harm can come from its use, furthermore if the nitrate of silver be not at hand at the birth of the child such simple remedies as lemon juice, alum, or even hot water may be applied very efficaciously. He quotes Professor Pinard, of Paris, who recommends the use of lemon juice as a prophylactic against ophthalmia neonatorum instead of nitrate of silver, being efficient, less irritating, always at hand, and handled without harm. If the disease has established itself, and, owing to the swelling of the conjunctiva the cornea is becoming impaired after the fourth or fifth day, Fox advocates eserin sulphate, gr. ¼—ʒ in addition to the ordinary nitrate of silver treatment. Speaking of atropin, which is as a rule in favour, Fox says, contrary to the reports made by other ophthalmic surgeons of the favourable results obtained by instillations of atropin, "I have failed to see one cornea saved after cloudiness appeared."

**Protargol**, which had such a boom for a little, now seems to be declining in favour. M. M. Fromaget and Degos[2] have published a series of cases in which it was used in a 1-10 ointment. They found it produced a rapid and permanent cure in cases of acute and subacute catarrhal conjunctivitis, but in purulent ophthalmia it was not so successful, and in phlyctenular ophthalmia quite useless and Stephenson, says, that judging from a discussion at the Ophthalmological Society of Paris, it appears on the whole to be inferior to nitrate of silver in trachoma and purulent ophthalmia, while most useful in acute conjunctivitis and vernal catarrh.

**Pemphigus of Conjunctiva.**—Dr. Bellencontre[3] reports an interesting case of this affection in a woman, aged 78, of cachectic appearance. When first seen she had been suffering from violent itching and pricking pain on the surface of the right eye for nine months. The lids were glued together for the greater part of the day The ocular conjuctiva was inflamed in its entire extent, the cornea was becoming vascular, though there were no ulcerations present, and the lachrymal openings were free. Vision had been failing for three months. The left eye showed merely a slight degree of inflammation of the conjunctiva. When first seen there was no eruption anywhere on the skin, though there were present on the legs the scars of old ulcerations. On the bucco-pharangeal mucous membrane there was a general redness over the whole surface, and on the soft palate there were some large round pale ulcers, surrounded by dark areolæ. The patient could not say whether she had ever had bullæ in the buccal cavity or not. The diagnosis of pemphigus of the conjunctiva was at once made, and was afterwards confirmed by the appearance of bullæ on the skin. The cicatricial contraction of the palpetral fissure continued while the patient was under observation in spite of varied forms of treatment, and the opening was eventually transformed into a narrow slit 8 m.m. long by 2 m.m. wide. The author considers that the explanation of the adhesions as the results of the cicatricial contractions in the ruptured bullæ is not logical. He points out that were this the case we should see true bullæ occasionally, as the number formed would have to be very considerable, and the production of the cicatricial contraction would be very irregular, corresponding to the formation of the bullæ. He considers that the disease in question shows many characteristics pointing to its being of trophic origin, and regards the shrinking of the conjunctiva rather as a symptom of pemphigus, concomitant with the bullæ on the skin, than as the result of the ephemeral bullæ on the conjunctiva itself.

**The Employment of Solutions of Toluidin Blue as a Collyrium and Area Stain for Corneal Abrasions and Ulcers.**—Vesey,[4] of Jefferson College, says of this new collyrium: "Toluidin blue is a member of the anilin group, closely related chemically to methylene blue." It appears as a bluish crystalline powder, readily soluble in alcohol and water. It never appears to have been used clinically so far. Merck and Co. manufacture it. The solution having been found to be quickly destructive of low forms of animal life in bacteriology, it was thought possible that it might be of use as a collyrium in some of the inflammatory conditions of the eye. Vesey has used it in his hospital for the last six months. The strength of the solution varied from 1-10,000 to 1-50, but the 1-1,000 strength seemed to be quite as efficient, and did not stain the skin so much as the stronger solution. The method of using it is, first, to flush out the conjunctiva sac with normal saline solution, or boric acid lotion, to get rid of the accumulated secretions, and then follow this by a thorough flooding with the toluidin blue solution. The stain has seemed to hasten materially the healing of indolent corneal ulcers, and to cause a marked diminution in the discharge in conjunctivitis, especially in the acute contagious and pneumococcus varieties, in which the discharge is so abundant. It has been of service in purulent dacryocystitis, the suppuration soon ceasing under its use, unless when diseased bone was present. The discoloration of the skin is easily wiped away with a moist pledget of cotton wool. An action of toluidin blue that is of considerable value is its power of staining corneal abrasions in a similar manner to fluorescin, only producing a dark blue instead of the light green fluorescin stain, and being more readily observed gives a greater contrast. The healthy cornea is left quite free from the stain. This staining power is of use in cleansing the *cul de sacs* whenever much discharge is present, as each little streak of mucous is stained a deep blue and its position at once located. Other anilin stains were tried for this purpose, but they all, except methylene blue, proved highly irritating.

**Removal of Steel by Electro-magnet.**—As to the advantage of this procedure, Dr. Edward Gault[5] says: Since the employment of the magnet in 1876 for the removal of fragments of steel, many eyes have been saved which formerly would have required excision. When the foreign body is located in the anterior chamber of the eye, or even in the lens, the prognosis is usually very good, both as regards the preservation of the eye and the retention of useful vision. When, however, the piece of steel has entered the vitreous, although it may in the majority of cases be removed, the results are disappointing, and are good only in a very small percentage of cases. Even though at first some vision is preserved, in the course of a few months it is often lost by detachment of the retina from shrinking of cicatricial bands which form in the wounded vitreous. In not a few cases, however, the eye retains its shape and outward appearance, and would not appear to be a source of danger to its fellow, according to Hirschberg, who, in 100 cases, has never met with sympathetic inflammation after magnet extraction. When it is remembered that until the employment of the magnet no good result could be expected unless the piece of steel became encysted in the eye; and that, Hirschberg says, during the ten years of his practice preceding the use of the magnet he had no successful case, the results, though not exhilarating, mark a great advance.

[1] Med. Bulletin, Vol. xxi., p. 41. [2] Jour. of Tropical Medicine, Mar., 1899, p. 224. [3] Brit. Jour. of Dermatology, April, 1899, p. 172. [4] Med. Chron., Feb., 1899, p. 389. [5] Inter-colonial Med. Jour., Feb. 20, 1899.

